# Changes in Olive Urban Forests Infected by *Xylella fastidiosa*: Impact on Microclimate and Social Health

**DOI:** 10.3390/ijerph16152642

**Published:** 2019-07-24

**Authors:** Teodoro Semeraro, Elisa Gatto, Riccardo Buccolieri, Marzia Vergine, Zhi Gao, Luigi De Bellis, Andrea Luvisi

**Affiliations:** 1 Department of Biological and Environmental Sciences and Technologies, University of Salento, 73100 Lecce, Italy; 2 School of Architecture and Urban Planning, Nanjing University, Nanjing 210093, Jiangsu Province, China

**Keywords:** ecosystem services, urban forest, phytopathology, numerical modelling, microclimate

## Abstract

This paper is devoted to the analysis of the impact of changes in olive urban forests affected by *Xylella fastidiosa* on ecosystem services. The focus is on microclimate and thermal comfort evaluated by two indices: the temperature of equivalent perception (TEP) and the predicted mean vote (PMV), which take into account both microclimate parameters and personal factors (heat resistance of clothing and human activity). The work has been carried out through (i) a qualitative analysis of the potential ecosystem services changes caused by temporary transition from olive groves to uncultivated soil, (ii) a study of the potential change of land use from monumental olive groves to other types of use, and (iii) a quantitative analysis on microclimate impact due to the loss of ecosystem services in two selected neighborhoods located in the Apulia region and chosen due to their proximity to the urban context. The analysis revealed that (i) direct effects on ecosystem services are principally linked with regulation functions and cultural services, (ii) a critical loss of cultural value of monumental olive groves occurred in the two neighborhoods, (iii) such a loss may lead to an increase of TEP and PMV, indicating a decrease of thermal comfort in the whole neighborhoods. Thus, it is necessary to plan the replanting policies of the use of the areas affected by *X. fastidiosa* not only in terms of agricultural planning but also in terms of landscape, urban planning, and human well-being.

## 1. Introduction

The global trend towards urbanization is determining the importance of the green component of the system that includes roads, car parks, footpaths, and other utility buildings. The social and environmental benefits of trees and woodlands are the result of an intricate array of processes that demonstrate the wide-ranging role that they play in the environment, economy, culture, and human well-being [1]. Several studies suggest that trees and green space positively affect people’s self-perceived health, determined by direct and indirect social and biological benefits such as increase of attention [2,3], reduction of stress [4], increase of life satisfaction [5] and positive emotions [6,7], lower mortality rates [8,9], and increase of physical activity [10].

An important element of urban green space is the urban forest, defined as a network or a system that includes all the woods, groups of trees, and individual trees located in urban and peri-urban areas. It, therefore, includes forests, road trees, trees in parks, gardens, and trees in abandoned corners [11]. The urban forests produce ecosystem services, reducing air, water, noise pollution and the urban heat island, working as natural air conditioners, preventing solar radiation from heating the buildings and surfaces, cooling the air by evapotranspiration and reducing wind speed [12,13,14].

In this context, the beneficial impact, as well as biodiversity conservation, increases if large old trees are considered [15]. Large old trees are among the biggest and most long-lived organisms on the earth. They greatly affect emotional perception and have many human cultural [16] and aesthetic values [17], as well as a key ecological role [18]. Large old trees also play a key ecological role and some studies have examined the important interactions on distribution, abundance, and interrelationships of many other elements such as water, nutrients, and organisms including fungi, other plants, and numerous species of animals [19]. Despite their fundamental impact on the life of the communities, these trees are declining globally [20]. Once the large old trees are lost from the community, some vulnerable ecosystems will collapse, and it will be hard to replace their cultural and ecological functions [21]. The real problem is that the large old trees will evolve far more slowly than most of their pathogens, such as microbes, fungi, bacteria, herbivores or wood-boring insects and vertebrate folivores. The negative impacts of invasive species are likely to be an increasingly important factor given the explosive rate at which foreign species are being introduced into ecosystems [22,23]. A classic example is the collapse of populations of the American chestnut (*Castanea dentata*) throughout the eastern USA as a result of the introduction of chestnut blight (*Cryphonectria parasitica*) from Asia, or the largest kauri trees (*Agathis australis*) in New Zealand, which are vulnerable to the effects of an introduced fungus-like pathogen (*Phytophthora agathidicida*) and for which there is presently no known effective treatment [24].

*Xylella fastidiosa* represents an emerging threat in Europe, since its finding at the end of 2013 in the Salento peninsula (Apulia region, southern Italy), associated to a previously unknown disease on olive trees (*Olea europaea* L.) which causes a leaf scorch (the so-called “olive quick decline syndrome”, OQDS), a rapid decline and the death of trees [25]. These symptoms are particularly severe on specifically large old plants of the most sensible cultivar “Cellina di Nardò” and “Ogliarola” [26,27,28], the most widespread in the whole area. The evidence and the following research studies indicated a connection with the bacterium *Xylella fastidiosa* subsp. *pauca* ST53 [29] (*X. fastidiosa* hereinafter). *X. fastidiosa* can be found worldwide, though its diseases are most prominent in America [30]. The further findings in France and Spain allow considering it as an expanding global pathogen [31], which could reshape not only agriculture but the landscape and human relations with plants. Indeed, the pathogen represents both an environmental threat (it is capable of infecting several woody plants and shrubs besides olive tree) and an economic plague (about 11 million of olive tree are planted in the Apulian infected area, while in the whole Mediterranean areas olive tree is among predominant cultivated species) [32]. Unfortunately, no cure is yet available and its control relies on uprooting infected trees and vector control. Furthermore, olive trees have a long historical and cultural link with the infected areas, and their demise causes a strong emotional impact on inhabitants and visitors [33].

Thus, the arrival of *X. fastidiosa* in Apulia has marked a political rupture due to the imposition of mandatory actions. This bacterium has threatened agriculture but also social and anthropological balances [33]. The conspicuous loss of an environmental, social-cultural, and economic heritage determined by this phytosanitary emergency prompts us to consider the fundamental interdisciplinary approach to understand, prevent, and reduce the harmful effects of invasive alien plant pathogens and pests in urban and peri-urban forests. At the same time, it is also crucial to evaluate the economic, environmental, and social loss, to foresee future scenarios and to be ready to implement policy actions aimed at mitigating such loss.

The aim of this study was the assessment of the human health benefits lost directly related to the devastating impact of the plant pathogen (*X. fastidiosa*) on olive trees. The study was divided into three steps:

The first step involved a qualitative analysis of the potential ecosystem services changes caused by the loss of olive trees through bibliographic research.

The second step was devoted to the study of the potential change of land use from olive groves to other types of use. Historical and current maps were employed. The social-cultural aspect of the loss of olive trees was also discussed.

In the third step, a quantitative analysis was performed to evaluate the impact on microclimate due to the loss of ecosystem services provided by olive trees in two transition areas (i.e., between urban boundaries and agricultural areas). This was assessed by air temperature and thermal comfort indices obtained through modelling simulations performed with the computational fluid dynamics and microclimate model ENVI-met. Three scenarios were considered: pre-epidemy (plants in good phytosanitary status), epidemy (actual status, with severe symptoms on plants) and a future scenario which considers the transition from olive trees in good health to uncultivated soil.

This study may provide useful indications in the planning of agricultural areas affected by *X. fastidiosa* that for their spatial location, ecological and social functions should be thought of as green spaces.

## 2. Study Area

**Figure 1:**
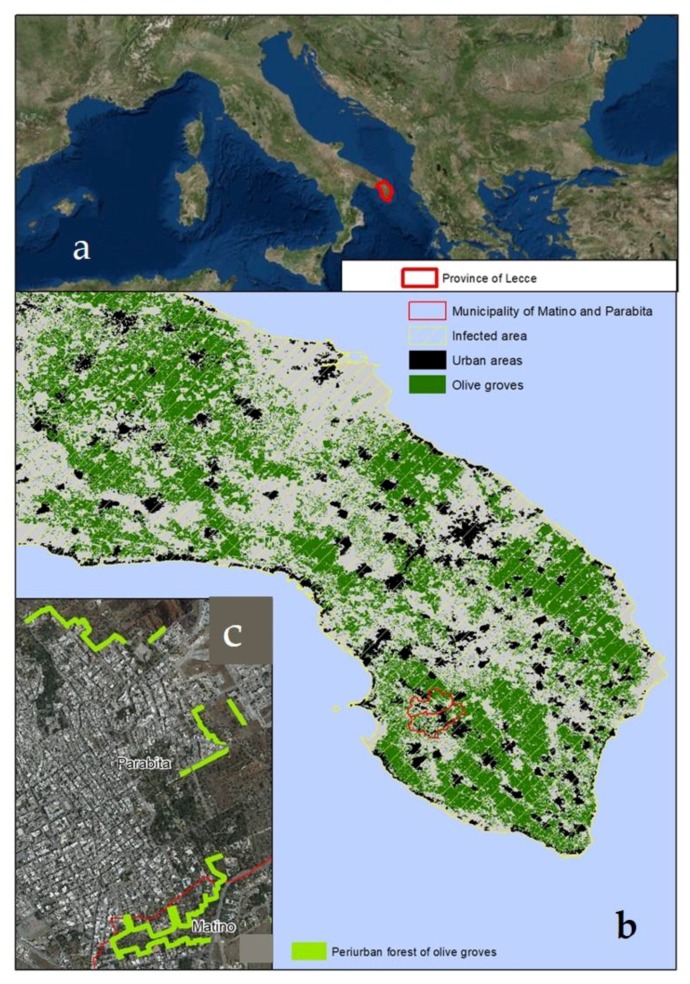
Study area: (**a**) The Province of Lecce in south Apulia; (**b**) the municipalities of Apulia region are characterized by a core urban mainly surrounded by olive groves, the latter currently strongly affected by *X. fastidiosa*, (**c**) the two selected peri-urban neighborhoods characterized by olive groves in the municipalities of Parabita and Matino. In the first case (municipality of Parabita) the olive groves surround the portion of the urban area, in the second case (municipality of Matino) the olive groves penetrate into the urban area.

The Province of Lecce located in southern Apulia (southern Italy) (Figure 1a) is characterized by the cultural landscape, which is the result of strong historical interactions between ecological, economic, and social components. The province extends for about 279,887 ha and about 41% of its extension is covered by olive groves. Unfortunately, many olives trees have been infected by *X. fastidiosa*, causing the desiccation and the death of infected plants, even if some resistant cultivars were identified [34,35]. The data retrieved in the institutional database (http://sit.puglia.it/portal/portale_gestione_agricoltura/Cartografie) confirm the presence of the pathogen in all the Lecce area, and the presence is considered as not eradicable [28].

Urban and rural areas are often interconnected, and a net separation is not evident. In these cases, peri-urban areas are characterized by agroecosystems [35,36]. Indeed, as shown in Figure 1b, many municipalities are characterized by a core urban area mainly surrounded by olive groves, the latter currently strongly affected by *X. fastidiosa*. The destructive effect caused by this pathogen on olive trees can potentially compromise the practical and ecological purposes of olive groves with direct and indirect effects on ecosystem services and effects on human health.

Two peri-urban neighborhoods characterized by olive groves in the municipality of Parabita (40°03′ N 18°08′ E, where almost 9047 people live in an area of 2109 ha) and Matino (40°02′ N 18°08′ E, where 11,444 people live in an area of 2800 ha) [37] were selected to assess the effect of their loss on the local population health, measured as loss or decrease in ecosystem services. These neighborhoods have been chosen due to their proximity to the urban context and representing the main spatial types of interaction between urban areas and olive groves. In the first case (municipality of Parabita), the olive groves surround the portion of the urban area, in the second case (municipality of Matino), the olive groves penetrate the urban area, to form an intrusion of the agroecosystem into the urban area (Figure 1c).

## 3. Materials and Methods

### 3.1. Ecosystem Services Changes

**Figure 2:**
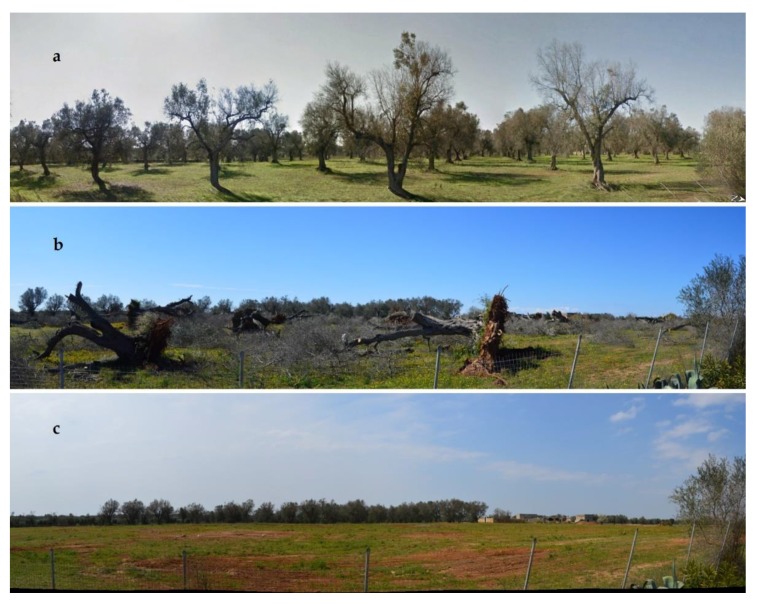
Example of eradication activity carried out in the early 2019. The scenarios represent the short- and medium-term transformation considered for the qualitative analysis of the change in ecosystem services caused by *X. fastidiosa*. (**a**) Picture of olive trees affected by *X. fastidiosa* (December 2018), (**b**) same as (**a**), but after eradication (February 2019), (**c**) same as (**a**) but some months after eradication (April 2019).

In order to evaluate the ecosystem service changes and, thus, the impact on human health caused by peri-urban areas land use transformation, a methodological analysis using the Environmental Impact Assessment (EIA) was followed, as indicated by the Directive 2001/42/EC of the European Parliament and of the Council of 27 June 2001, commonly applied to a wide range of public plans and programs (e.g., land use, transport, energy, waste, agriculture, etc.). The alterations which occurred in the main environmental matrices (air, soil, water, landscape, biodiversity) were selected from the literature (see Table 1 caption later in the text) and applied to likely scenario (from olive groves to uncultivated soil) following the implementation of the regulation of the Apulia region of 5 February 2019 which requires the felling of trees infected with *X. fastidiosa* (Figure 2).

### 3.2. Changes in Olive Grove and Disease Severity Assessment

First, the olive groves maps of years 1887 and 2016 have been digitized and analyzed using the Geographic Information System software QGIS (qgis.org). For 1887, the historical map of land use carried out by the milizia forestale was used. The map of the olive groves for years 2016 was downloaded in shapefile format from the official Territorial Information System (SIT) of the Apulia Region (www.sit.puglia.it/). After that, a change detection analysis was carried out overlapping olive groves maps of 1887 and 2016 to identify potential old olive groves in the Apulia region. Such a long timespan of the land-use analysis has been chosen to define the potential spatial distribution of monumental olive groves in the Salento area.

The cultural value of monumental olive groves was then analyzed through objective measures concerning the diameter of the trunk and through a visual analysis of the shape of the trunk of the plants as established by the regional law 14/2007 (B.U.R. PUGLIA—N. 83, 07/06/2007). Symptoms of *X. fastidiosa* related to leaf scorching and wilting of the canopy were assessed by visual inspection, estimating disease severity and using the following severity scale: 0 = symptomless, 1 = leaf scorching on few branches or few desiccated branches affecting the portion of the canopy, 2 = leaf scorching on several branches or desiccation affecting a large part of the portion of the canopy, and 3 = canopy with desiccated branches uniformly distributed [32].

### 3.3. Impact of Olive Tree Loss on Microclimate in the Selected Neighborhoods

**Figure 3:**
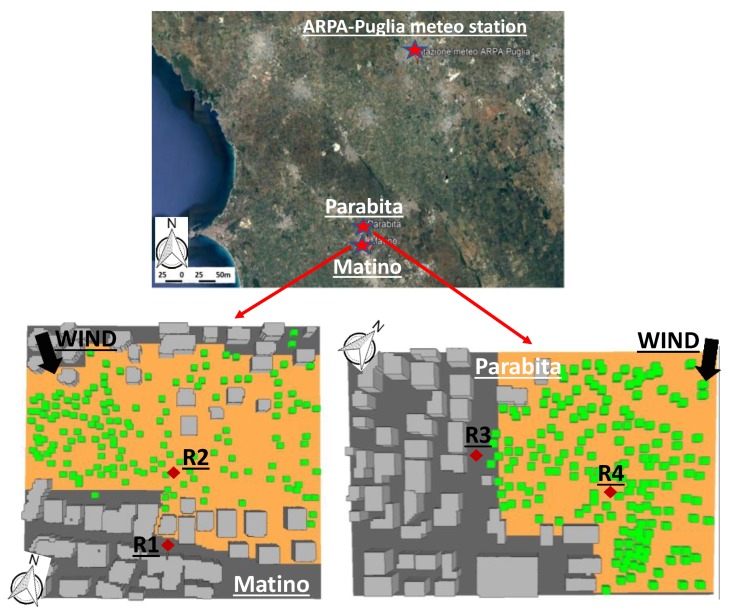
Geographical position of the ARPA-Puglia meteorological station with indication of the study neighborhoods (Matino and Parabita) (from Google Earth) and their reconstructions in ENVI-met (red rhombus indicate the receptors where data have been extracted for microclimate analysis).

As mentioned in Section 2, the two neighborhoods analyzed are located in Matino and Parabita, two small-size cities of south Italy characterized by a Mediterranean climate and a classical Mediterranean architectural design, consisting of two-to-three-story buildings and narrow street canyons (Figure 3). The neighborhoods are characterized by buildings heights ranging from 7 to 11 m (heights estimated using the DISTO) and by the presence of about 155 olive trees in Matino and 170 in Parabita.

The leaf area index (LAI) of tree crowns was estimated from measurements of the photo-synthetically active radiation (PAR) acquired by an Accu-PAR LP80 ceptometer. All measurements were taken parallel to the ground. Five replicas were done just near the crown (where the sensor measured unobstructed PAR) and at its base (where it was supposed that the LAI is maximum). The leaf area density (LAD, m^2^m^–3^) was thus estimated dividing LAI by the depth of the tree crown. The analysis allowed to characterize the infected olive groves having a height of 8 m and a crown depth of 3 m, crown height of 4 m, and a leaf area density (LAD) of 0.3 m^2^m^–3^. Olive groves in good health have the same height, but a depth crown of 5 m and a LAD of 1.1 m^2^m^–3^.

Three scenarios have been modeled: (i) the current scenario with olive groves affected by *X. fastidiosa* (epidemy scenario), (ii) a scenario with plants in good phytosanitary status (pre-epidemy scenario), and (iii) a scenario with no olive trees representing a transition to an uncultivated soil (future scenario).

#### 3.3.1. Numerical Simulations

ENVI-met is a prognostic non-hydrostatic model for the simulation of surface-plant-air interactions composed by a 3D main model and, in addition, a one dimensional (1D) atmospheric boundary layer (ABL) model which extends from the ground surface up to 2500 m. ENVI-met has a typical horizontal resolution from 0.5 to 5 m and a typical time frame of 24 to 48 h with a time step of 1 to 5 s, which meet the criteria for the accurate simulation of physical processes, suitable for microclimate studies at the neighborhood scale. The atmospheric system solves Reynolds-averaged Navier-Stokes equations using a 1.5 order turbulence closure *k-ε* model. Equations related to surfaces thermal exchanges are calculated from an energy balance of the net radiative energy fluxes, turbulent fluxes of heat and vapor and soil heat flux. Evapotranspiration processes are simulated during the entire daily cycle on the basis of the biological and thermo-physical properties of plants and soils. Several studies have shown that ENVI-met is able to simulate both spatial and temporal temperature and wind speed for the evaluation of microclimate in both simple and complex urban areas. The recent review by [38] provides evidence of its suitability for the cases studied in this paper. See also [39] for a review of validation studies.

The 3D simulation area (computational domain) of the Matino neighborhood has a dimension of 300 m (x-direction) × 300 m (y-direction) × 60 m (z-direction), while the area of the Parabita neighborhood is 300 m × 200 m × 60 m. The areas were meshed using a regular grid with a resolution of 2 m × 2 m × 2 m, except for the lowest (close to the ground) five cells whose vertical resolution was 0.4 m (i.e., the equidistant option was employed in ENVI-met). To improve model accuracy and stability, five nesting grids were also employed. The cyclic type method was selected for the lateral boundary conditions (LBC) because the study area is located in the city where the neighborhood space layout is similar to the study area. Hourly air temperature and relative humidity were forced at the model boundary to drive the simulation with meteorological input of 18 August 2018, selected as representative of a typical summer day, since the temperature profile was reasonably correlated with the average temperature profile, and the wind (speed = 1.56 m/s; direction = 329°) was rather stationary and consistent with the prevailing wind during the period July–August 2018. Data have been obtained from a 10 m high ARPA-Puglia (the Regional Agency for Environmental Protection of the Apulia region) meteorological station (Figure 3), located at about 20 km from the neighborhoods (http://www.arpa.puglia.it/web/guest/serviziometeo). The minimum and maximum temperatures were 22.1 °C and 32.9 °C, while the minimum and maximum relative humidity were 44% and 87%. Wind speed was set to 1.56 m/s (mean daily value) and wind direction to 329° (median of wind directions during the day). For each case, ENVI-met was run for a 16 h period, starting at 06:00.

#### 3.3.2. Thermal Comfort Indices

Together with air temperature, the impact of olive groves and their loss on thermal comfort was evaluated by means of the predicted mean vote (PMV) and the temperature of equivalent perception (TEP). PMV (-) allows for the evaluation of the thermal sensation based on the heat balance of the human body. It takes into consideration the air temperature, the mean radiant temperature (MRT), the wind speed and the relative humidity, as well as the personal factors, e.g., heat resistance of clothing and human activity [40]. The outdoor PMV range and the corresponding human thermal sensation are: 4 (very hot), 3 (hot), 2 (warm), 1 (slightly warm), 0 (neutral), −1 (slightly cool), −2 (cool), −3 (cold), −4 (slightly cold). TEP (°C) is sensitive to changes in air temperature and MRT [41]. Personal factors such as clothing insulation and the level of activity are not considered (as in PMV). The thermal perception ranges are: ≥42.5 °C (very hot), 34.9–42.4 °C (hot), 27.3–34.8 °C (warm), 19.6–27.2 °C (neutral), 12.0–19.5 °C (cool), 4.4–11.9 °C (cold), <4.3 °C (very cold).

Temperature, PMV, and TEP were calculated based on parameters directly obtained from ENVI-met. The data were extracted at receptors indicated in Figure 3 to evaluate the influence of olive trees within the olive grove and in nearby street canyons. The selected height for the calculations was 1.4 m (pedestrian height).

## 4. Results

### 4.1. Analysis of Ecosystem Services Changes

The whole Province of Lecce has been classified as infected by the bacterium and the completely compromised agroecosystem is causing a temporary transition from olive groves to uncultivated soil. It should be also noted that a regional law (5 February 2019) indicated the removal of infected trees within the infected areas [42], and, thus, it is expected that the Province of Lecce will have a sudden change in the landscape pattern with direct effects on the ecosystem services linked principally with the regulation functions like CO_2_ and climate regulation (Table 1). In particular, the CO_2_ sequestered per century-old tree is estimated to be about 2600 kg CO_2_ which could be reintroduced in the atmosphere if all the wood produced by the explant of the trees were disposed of by burning. However, these changes can be considered reversible in the medium-to-long term, if the areas of monumental olive trees are be replaced by other arboreal plants that may constitute the future forests of the Salento. It is possible to consider that in 30–40 years, the planting of arboreal plants could restore the ecological functions lost due to the loss of the historic olive groves.

Other important aspects linked to the loss of ecosystem services are related to agricultural heritage information and functions (Table 1). Although the importance of the monumental olive groves on the historical and cultural value of the Salento landscape is certified by the regional law (14/2007), this aspect is difficult to quantify. These changes can be considered irreversible over time because there are no longer social, economic, and environmental conditions that can lead to the development of a historic olive grove as it is known now. These effects could be felt more in urban areas where there is a large number of individuals who benefit every day from the relations existing between peri-urban forests consisting of historic olive groves and the well-being of the population.

**Table 1:** Functions, goods and services identified for natural and semi-natural ecosystems [43,44,45,46,47] integrated with the potential variation of such services connected with the change of land use from old olive groves to uncultivated land [48,49,50,51,52,53,54,55,56,57,58,59,60,61]. The emoticon ☺ indicates positive alteration, ☹ indicates negative alteration, whereas the symbol = indicates no significant alteration. The number of emoticons gives an estimate of the intensity of the alteration: 1: low alteration; 2: medium alteration; 3: strong alteration.

Functions		Ecosystem Processes and Components	Ecosystem Services	Variation from Old Olive Groves to Uncultivated Areas	Environmental M atrix Directed and Undirected Affected	Reversible
**Regulation functions** (Maintenance of essential ecological processes and life support systems)	Gas regulation	Role of ecosystems in bio-geochemical cycles (e.g., CO_2_/O_2_ balance, ozone layer, etc.)	UVB-protection by O_3_ (preventing disease); Maintenance of (good) air quality; Influence on climate	☹☹☹	Air Biodiversity population	Yes
	Climate regulation	Influence of land cover and biol. Mediated processes (e.g., DMS-production) on climate	Maintenance of a favorable climate (temp., precipitation, etc.) for human habitation, health, cultivation.	☹☹	Air Soil Water Biodiversity population	Yes
	Disturbance prevention	Influence of ecosystem structure on dampening env. disturbances	Storm protection (e.g., by coral reefs); Flood prevention (e.g., by wetlands and forests)	not evaluated		
	Water regulation	Role of land cover in regulating runoff and river discharge	Drainage and natural irrigation	=	Soil Water Biodiversity population	
	Water supply	Filtering, retention and storage of fresh water (e.g., in aquifers)	Provision of water for consumptive use (e.g., drinking, irrigation and industrial use)	=	Soil Water Biodiversity population	
	Soil retention	Role of vegetation root matrix and soil biota in soil retention	Maintenance of arable land; Prevention of damage from erosion/siltation.	☹	Soil Biodiversity population	Yes
	Soil formation	Weathering of rock, accumulation of organic matter	Maintenance of productivity on arable land; Maintenance of natural productive soils;	☹	Soil Biodiversity population	Yes
	Nutrient regulation	Role of biota in storage and recycling of nutrients (e.g., N, P and S)	Maintenance of healthy soils and productive ecosystems	☹	Soil Water Biodiversity population	Yes
	Waste treatment	Role of vegetation and biota in removal or breakdown of xenic nutrients and compounds	Pollution control/detoxification; Filtering of dust particles (air quality) Abatement of noise pollution	not evaluated	Soil Water Biodiversity population	
	Pollination	Role of biota in movement of floral gametes	Pollination of wild plant species; Pollination of crops.	=	Biodiversity population	
	Biological control	Population control through trophic-dynamic relations	Control of pests and diseases; Reduction of herbivory (crop damage).	not evaluated	Soil Biodiversity population	
**Habitat functions** (Providing habitat (suitable living space) for wild plant and animal species)	Refugium function	Suitable living space for wild plants and animals	Maintenance of biological and genetic diversity (and, thus, the basis for most other functions)	☹	Biodiversity Population cultural landscape	Yes
	Nursery function	Suitable reproduction-habitat	Maintenance of commercially harvested species	☹	Biodiversity Population cultural landscape	Yes
**Production functions** (Provision of natural resources)	Food	Conversion of solar energy into edible plants and animals	Hunting, game, fruits, etc. Small-scale subsistence	☹	Biodiversity Population cultural landscape	Yes
	Raw materials	Conversion of solar energy into biomass for human construction and other uses	Building and Manufacturing (e.g., lumber); Fuel and energy (e.g., fuel wood);	☹	Population cultural landscape	Yes
	Genetic resources	Genetic material and evolution in wild plants and animals	Improve crop resistance to pathogens and pests; Other applications (e.g., health care)	not evaluated		Yes
	Medicinal resources	Variety in (bio)chemical substances in, and other medicinal uses of, natural biota	Drugs and pharmaceuticals; Chemical models and tools; Test and essay organisms	not evaluated		Yes
**Information functions** (Providing opportunities for cognitive development)	Aesthetic information	Attractive landscape features	Enjoyment of scenery (scenic roads, housing, etc.)	☹☹☹	Population cultural landscape	No
	Re-creation	Variety in landscapes with (potential) re-creational uses	Travel to natural ecosystems for eco-tourism and (re-creational) nature study	☹☹☹	Population cultural landscape	No
	Cultural and artistic information	Variety in natural features with cultural and artistic value	Use of nature as motive in books, film, painting, folklore, national symbols, architect	☹☹☹	Population cultural landscape	No
	Spiritual and historic information	Variety in natural features with spiritual and historic value	Use of nature for religious or historic purposes (i.e., heritage value of natural ecosystems and features)	☹☹☹	Population cultural landscape	No
	Science and education	Variety in nature with scientific and educational value	Use of natural systems for school excursions, etc. Use of nature for scientific research	☹☹☹	Population cultural landscape	No
**Carrier functions** (Providing a suitable substrate or medium for human activities and infrastructure)	Habitation	Depending on the specific land use type, different requirements are placed on environmental conditions (e.g., soil stability and fertility, air and water quality, topography, climate, geology, etc.	Living space (ranging from small settlements to urban areas)	☹☹☹	Population cultural landscape	No
	Tourism-facilities		Tourism-activities (outdoor sports, beach-tourism, etc.)	☹☹☹	Population cultural landscape	No

### 4.2. Analysis of Changes in Olive Grove and Disease Severity Assessment

**Figure 4:**
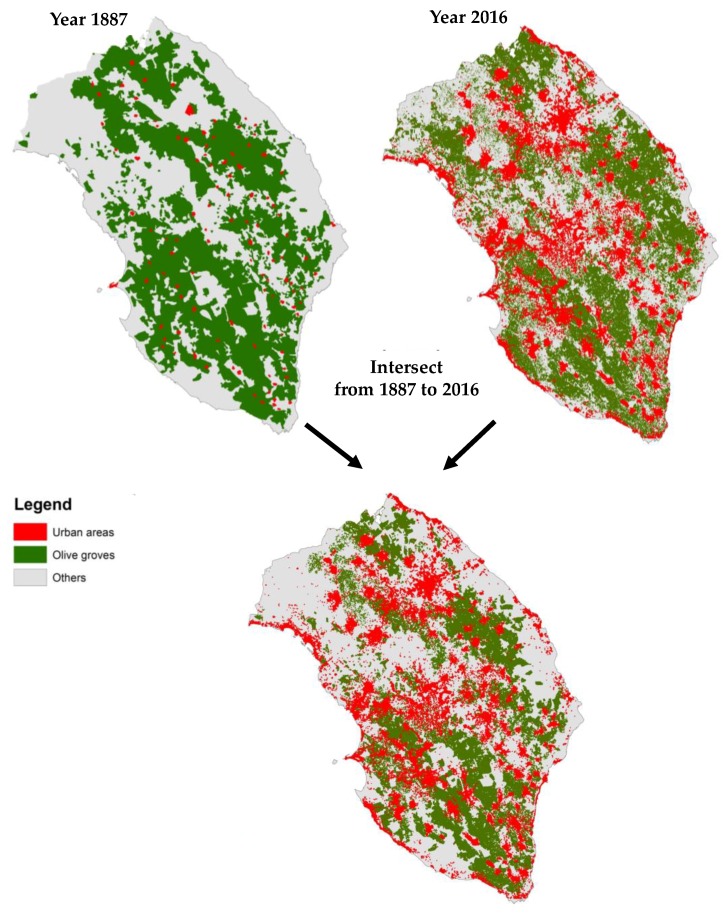
Maps showing the presence of urban areas, olive groves and other land uses in the years 1887 and 2016 and relative intersection.

The maps (Figure 4) revealed that 150 years ago the olive grove covered about an extension of 124,138 ha, which was about 44% its extension. Around 66,475 ha of olive groves can potentially be classified as historical, because they are shown on the maps of 1887 and 2016. In this sense, the monumental olive grove represents 58% of the olive groves currently present and about 24% of the total extension of the Province of Lecce. Therefore, old olive groves represent a structural element in the pattern of the province, and many olive trees are secular plants with a high cultural value recognized and protected by the Apulia regional law 14/2007.

**Figure 5:**
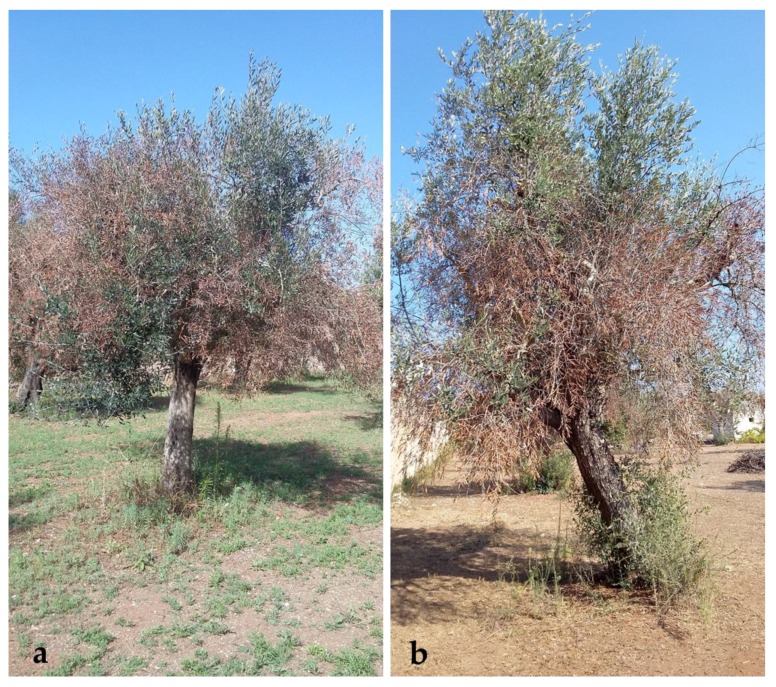
Olive trees studied in municipalities of (**a**) Matino and (**b**) Parabita with symptoms of disease (“olive quick decline syndrome”, OQDS) severity between level 2 to 3.

From a field survey, it emerged that over the 51% of olive groves located in the two study neighborhoods are characterized by monumental plants, therefore, they can be considered historic olive groves. The trees present a sculptural shape like defined in the regional law (14/2007) and trunk size is greater than 100 cm of diameter. This confirms what was found in the previous analysis of the olive grove change. These old olive groves are an important element of the cultural urban area of Matino and Parabita. However, as visible in Figure 5, most plants have the symptoms of desiccation caused by *X. fastidiosa*, with a disease severity esteemed between levels 2 to 3 out of 3. Therefore, they are subjected to decay or death.

### 4.3. Analysis of the Impact of Olive Tree Loss on Microclimate in the Selected Neighborhoods

#### 4.3.1. Temporal Profiles of Air Temperature, TEP and PMV

**Figure 6:**
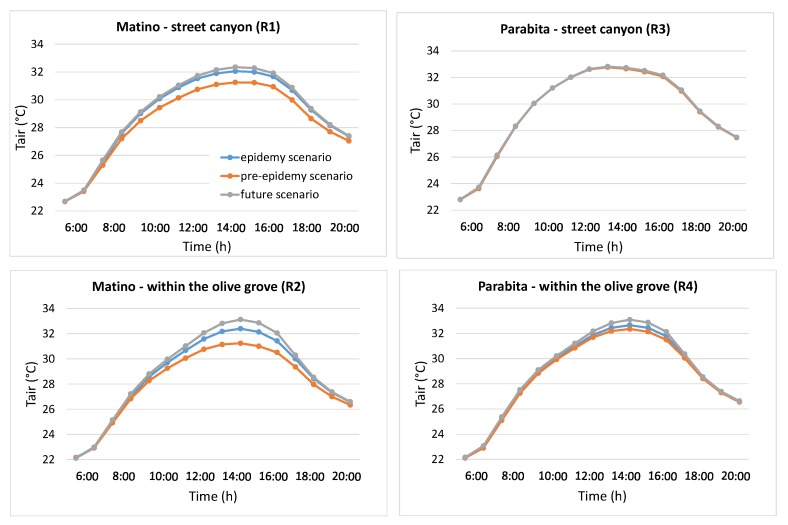
Air temperature hourly profiles for Matino (left) and Parabita (right) inside the street canyon (top) and within the olive grove (bottom) (see Figure 3 for the position of receptors R1, R2, R3 and R4).

**Figure 7:**
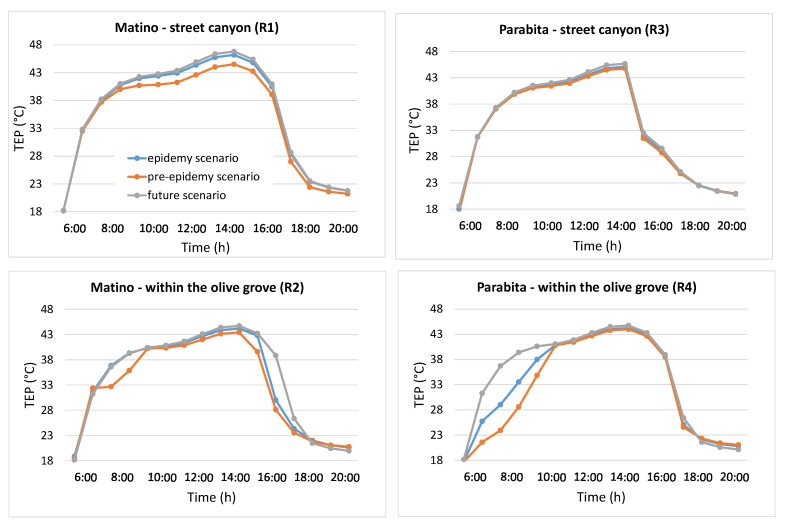
Temperature of equivalent perception (TEP) hourly profiles for Matino (left) and Parabita (right) inside the street canyon (top) and within the olive grove (bottom) (see Figure 3 for the position of R1, R2, R3 and R4 receptors).

**Figure 8:**
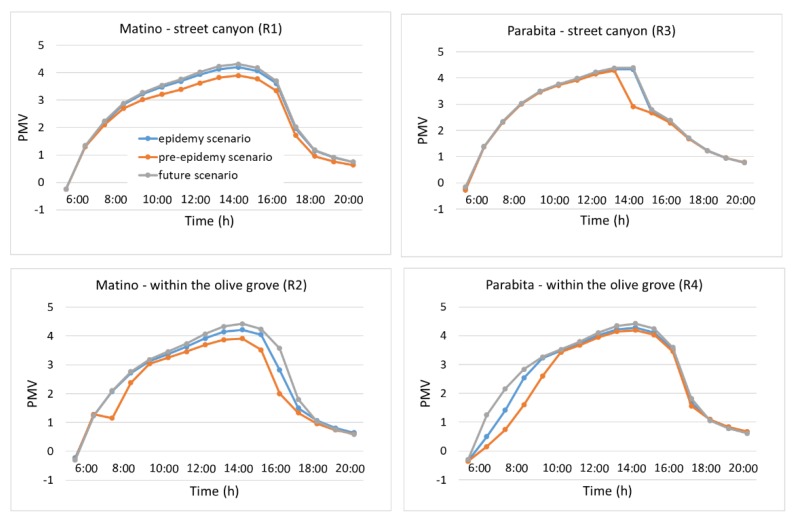
Predicted mean vote (PMV) hourly profiles for Matino (left) and Parabita (right) inside the street canyon (top) and within the olive grove (bottom) (see Figure 3 for the position of R1, R2, R3 and R4 receptors).

As suggested from Table 1, the variation from old olive groves to uncultivated soil (uncultivated areas) is expected to affect their climate regulation and thus the maintenance of a favorable climate in terms of temperature, humidity, etc. for human health. In order to quantitatively evaluate such impact, temporal profiles of air temperature, TEP and PMV, at the receptors located inside the street canyon and within the olive groves for the two selected neighborhoods are shown in Figure 6, Figure 7 and Figure 8.

Figure 6 shows that in both neighborhoods the air temperature (Tair) in the future scenario (i.e., uncultivated soil without olive trees) is higher than that in the other two scenarios (epidemy and pre-epidemy) during all day, and especially during the central and hottest hours of the day (10:00–17:00). As expected, the presence of trees leads to a decrease of air temperature, even though the effect is small [32], and it is larger with olive trees in good phytosanitary status (pre-epidemy scenario) due to larger shading and evapotranspiration effects.

Further, it is possible to observe that, due to the different configuration of the olive groves and the geometry of the neighborhoods, the effect of the presence of trees (in terms of decrease of air temperature) is more evident in Matino, and the largest decreases for all cases occur at 15:00. In particular, the street canyon in Matino shows a decrease of 0.30 °C in the epidemy scenario and 0.80 °C in the pre-epidemy scenario, while within the olive grove a decrease of 0.75 °C and 1.15 °C, respectively, occur. On the other hand, Parabita shows only a decrease within the olive grove (of 0.40 °C and 0.70 °C, respectively), while no effect of the trees is observed in the street canyon, likely due to the distance of the canyon from the olive grove location.

To evaluate the effect on thermal comfort, Figure 7 and Figure 8 show that TEP and PMV decrease in the presence of trees, suggesting an improvement of thermal comfort. Different from the air temperature, where the profiles follow the same behavior, here TEP and PMV profiles show substantial differences between the two receptors (i.e., in the street canyon and within the olive grove) and during the day, since the thermal comfort (here in terms of TEP and PMV) depends not only on the air temperature but also on relative humidity, wind speed, and MRT.

Specifically, for TEP profiles inside the street canyon in Matino, a decrease during the central hours of the day (9:00–16:00) is observed, with a maximum of 0.61 °C in the epidemy scenario and 2.35 °C in the pre-epidemy scenario at 14:00. Within the olive grove there is a very large decrease during the morning (3.95 °C at 8:00) and the afternoon (up to more than 10 °C at 17:00) between the pre-epidemy and future scenarios, but this is due to the shading effect of trees and, thus, to its effects on decreasing MRT in that particular receptor location. A similar trend is observed for PMV with the largest decrease at 15:00 in the street canyon (equal to 0.42 in the epidemy scenario and 0.11 in the pre-epidemy scenario) and at 17:00 within the olive grove (equal to 0.75 in the epidemy scenario and 1.57 in the pre-epidemy scenario).

In the case of Parabita, the main differences (for both TEP and PMV) are found within the olive grove during the first hours (7:00–10:00), with the largest decrease of more than 12 °C for TEP and 1.41 points for PMV at 8:00. As mentioned before, this is caused by the shading effect of trees which lead to a low MRT at the receptor location.

#### 4.3.2. Spatial Distribution of PMV

**Figure 9:**
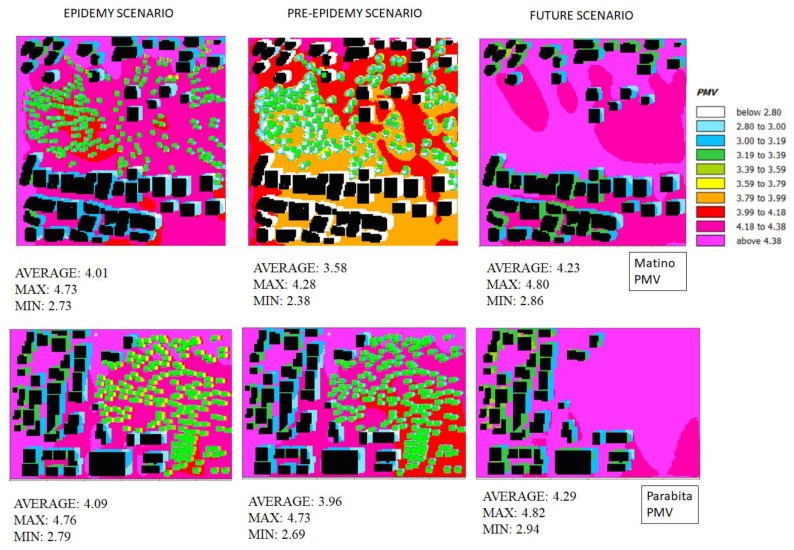
Spatial distribution of PMV at 15:00 for Matino (top) and Parabita (bottom) inside the street canyon (top) and within the olive grove (bottom) (see Figure 3 for the position of receptors R1, R2, R3 and R4).

Finally, Figure 9 shows 1.4 m high spatial contours of PMV in the afternoon (at 15:00). As expected, since the simulations were performed on 18 August 2018, a typical hot summer day, all the maps show an uncomfortable condition (from hot to very hot, see Section 3.3.2). In both neighborhoods, the presence of olive groves in good health (pre-epidemy scenario) induces an overall improvement of the thermal comfort (i.e., a decrease of PMV), while the PMV distribution of the current (epidemy) and future (uncultivated soil) scenarios are similar. Specifically, in Matino, the presence of olive grove leads to an average PMV reduction of 0.22 in the epidemy scenario and 0.65 in the pre-epidemy scenario, while in Parabita, the average PMV reduction is equal to 0.20 and 0.33, respectively.

It is worth noting that, as already mentioned before and suggested by the profiles of Tair, TEP, and PMV, the overall effect is more evident in Matino and the pre-epidemy scenario with an extra impact not only close to the trees but also in nearby empty street canyons.

## 5. Discussion

The present study represents one of the first analyses of the impact of urban olive groves on ecosystem services, and, in particular, on microclimate regulation. The study area comprises the municipalities of Matino and Parabita (Province of Lecce, Italy), which together represent a wide range of urban forms present in the social-ecological landscape of the Apulia region.

The old and monumental olive groves in the Province of Lecce are characterized by a tree structure with an average height of 5–8 m and a crown diameter of 3–5 m and characterized by a high LAI, so their widespread presence in the territory configures them as a forest. They are ecosystem service providers, due not only to the production of ecosystem goods (olives and oil), but also to their ecological role of sink biodiversity, which seems to simulate the role of forests in facing disturbances across spatial and temporal scales [62].

The severity of the problem of OQDS in Apulia is characterized by the fact that most of the olive trees in the region are monumental and thus cherished for their ecological and cultural value. The bacterium *X. fastidiosa* caused, up to now, an incurable disease, and the only available solution to reduce the spread of both the pathogen and its insect vectors are the elimination of diseased plants and control measures of the vector. Unfortunately, the phytosanitary policies have been largely criticized and strongly opposed by some local administrators and by organized social and environmental movements, causing a *de facto* spread of the bacterium at the local and regional scale, with economic and cultural impacts on the landscape. The spread of *X. fastidiosa* will produce a conversion of land use in the short-to-medium term leading to a reduction in ecosystem services and thus introduce critical issues. In particular, the case studies of Matino and Parabita have shown that the current scenarios of olive groves are close to uncultivated soil scenario characteristics in terms of microclimate regulation and, in particular, of thermal comfort. For example, the high concentration of dry biomass linked to the mortality of olive groves near urban areas is expected to increase potentially damaging fires [61,63], while the cutting of dry trees will produce a high emission of CO_2_ in the atmosphere due to the destruction of the wood mass and less absorption of CO_2_ with direct effects on population health.

Local management practices have resulted in distinctive cultural landscapes adapted to the specific climate and geographic areas, and old olive groves are the result of continuous interactions between nature and human activities [60,61]. The current state of the olive groves in the Province of Lecce derives from a type of tree cultivation aimed at favoring the manual harvesting of olives. The sculptural form of the trunk, on the other hand, is the result of plant maintenance actions aimed at eliminating the rotten wood of the trunk. The planting of new olive groves will adapt to the mechanical harvesting of olives and, therefore, to types of trees with short stems. Moreover, there will be a greater turnover of single trees to always maintain high productivity. The destruction of the old olive groves could mean the loss of the historical-cultural and natural heritage that cannot be recovered. Therefore, the process leading from agroforestry to cultural landscape must be considered in the land use transformation in peri-urban areas, not only for agricultural production but also for major contributions in the heritage of the peri-urban area on the wellbeing of the population [18].

In particular, the conversion of the monumental olive groves into uncultivated areas in the context of urban areas could also cause a worsening of the soundscape linked to the presence of fauna linked to the old olive groves. Therefore, old olive groves in the peri-urban area may be considered of high nature and cultural value (HNCV) green urban spaces that integrates woody vegetation with crops activities, and which are valued for their biodiversity, ecosystem services support, and their cultural heritage [64].

Thus, it is necessary to undertake planning actions devoted to the construction of new peri-urban landscapes and keeping important ecosystem services to improve the quality of human life and well-being. New landscape policies able to improve the adaptability and the capacity of actors in a system to influence and manage resilience is thus required [65]. In the case of old olive groves in peri-urban area, the adaptability could be interpreted like the capacity of the actors to develop a new system because the ecological, economic, or social (including political) conditions make the existing system untenable [65,66], but keeping the main ecological functions able to produce ecosystem services supporting wellbeing of the people that live in peri-urban area. Therefore, the adaptability and transformability, in this way, are not in contrast but complementary. Naturally, in a social-ecological system, the policy must considerate the panarchy because of cross-scale interactions, the resilience of a system at a particular focal scale will depend on the influences from states and dynamics at scales above and below. For example, external oppressive politics, invasions, market shifts, or global climate change can trigger local unexpected changes [65].

An obstacle in trying to improve the adaptability and transformability in the peri-urban forest in Matino and Parabita is the strong fragmentation of property. This situation also impedes the management of olive groves affected by *X. fastidiosa*, because of multiple notifications for uprooting area being requested, and proprieties of olive groves are not always easily assertible. Owners that do not have an agricultural income may show little interest in investing money to recreate a new urban forest with the intent of producing the same ecosystem services as the old olive groves.

**Figure 10:**
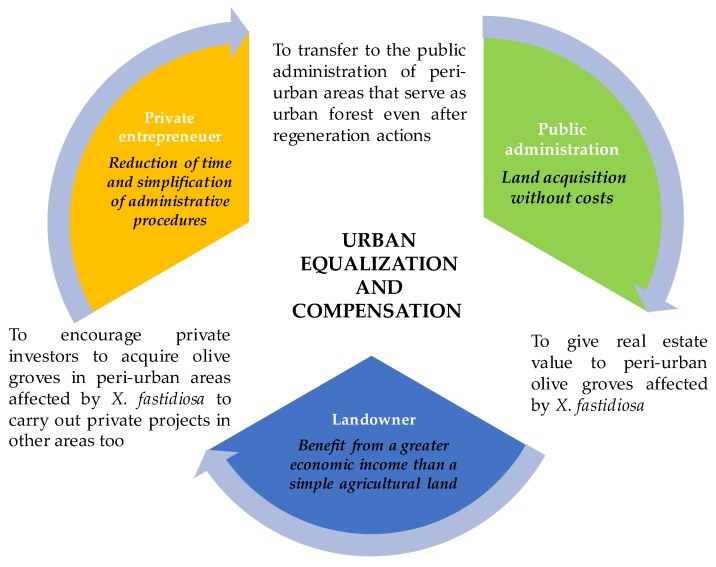
Conceptual model of management of areas affected by *X. fastidiosa*.

In the future, it will be necessary to develop territorial governance action or policies that will have to combine with the different needs of many stakeholders of the territory, or worse, with the limited interest of the stakeholders in investing economic resources and time in the landscape [36]. In particular, in areas adjacent to the urban context, such as those of Matino and Parabita investigated here, the problem of *X. fastidiosa* should also be managed through urban planning policies and not only considering agricultural policies. Our numerical results have shown that the trees have an effect on thermal comfort where they are located, but the effect can spread over the whole neighborhood and, thus, it is crucial to plan their planting within appropriate urban development Ppans. An urban planning tool useful for managing these areas could be the Equalization and Urban Compensation (Apulia regional law 27/07/2001) (Figure 10).

## 6. Conclusions

This study evaluates the impact of the loss of olive groves due to the plant pathogen *X. fastidiosa* in the Apulia region, with a focus on the Province of Lecce. *X. fastidiosa* is an expanding global pathogen which can be found worldwide, as confirmed by further findings in France and Spain. In this study, its effects have been evaluated in two neighborhoods of the province (located in the small towns of Matino and Parabita), selected due to their proximity to the urban context and representing the main spatial types of interaction between urban areas and olive groves in the province. The rationale behind this study is that olive trees play a key role in the urban environment of the Apulia region, not only from the agronomical and social-cultural point of views, but also because they improve the microclimate and mitigate the effects of climate change and Urban Heat Island. The present study has confirmed the impact of disease severity and has shown that the transition from olive trees in good health status to uncultivated soil has an effect on microclimate and, in particular, on thermal comfort. This impact is not just limited to places where trees are located (at the boundaries of the two neighborhoods) but in the whole region. Thus, it is crucial to study their effects and plan their planting within appropriate urban planning strategies to maximize their benefits. In this sense, the current agricultural legislation provides for the restoration of the olive grove made up of *X. fastidiosa*-resistant cultivars (two of them have been defined [34], while research continues to find others), at the same time it would be desirable to avoid a monovarietal approach and thus, to increase the ecological resilience to this and other pathologies.

The replanting policies of the olive groves or of the use of the areas affected by *X. fastidiosa* must, thus, be addressed not only in terms of agricultural planning but also in terms of landscape and urban planning, to avoid the abandonment of these areas due to the lack of owners’ interest in investing in a new olive grove. This requires a transdisciplinary approach that goes from plant physiology to urban planning and is able to identify and correlate the interactions between ecological and anthropic processes and between the different ecological scales and institutional levels, allowing the development of a holistic vision of the evolution of the landscape. Although providing public-health benefits are not the primary focus of many urban-forestry programs, in this specific case strategic planning is needed, which should not be limited to simply replacing sick plants with new plants, but that should be able to encourage the development of a new landscape able to provide similar ecosystem services provided by old olive groves.

The Apulia region has started a new challenge, not just focusing on what the territory is losing, but on what it can be gained from this situation. This implies a change of approach to try to be more proactive and not just conservative.
